# Does aging affect the immune status? A comparative analysis in 300 healthy volunteers from France, Austria and Spain

**DOI:** 10.1186/1742-4933-10-38

**Published:** 2013-09-06

**Authors:** Marie-Paule Vasson, Marie-Chantal Farges, Nicolas Goncalves-Mendes, Jérémie Talvas, Josep Ribalta, Brigitte Winklhofer-Roob, Edmond Rock, Adrien Rossary

**Affiliations:** 1 Clermont Université, Université d’Auvergne, Unité de Nutrition Humaine, F-63000, Clermont-Ferrand, France; 2 INRA, UMR, 1019, Equipes ECREIN & MICROCARD, CRNH Auvergne, F-63009, Clermont-Ferrand, France; 3 Centre Jean Perrin, CHU Clermont-Ferrand, Service de Nutrition, F-63000, Clermont-Ferrand, France; 4 Unitat de Recerca en Lípids i Arteriosclerosi, Facultat de Medicina, Hospital Universitari de Sant Joan de Reus, Universitat Rovira i Virgili, Institut d’Investigació Sanitària Pere Virgili, CIBERDEM, Reus, Spain; 5 Human Nutrition & Metabolism Research and Training Center (HNMRC), Institute of Molecular Biosciences, Karl-Franzens University, Graz, Austria

**Keywords:** Aging, Immune status, Europe

## Abstract

**Background:**

As the European population is getting older, there is growing need in scientific data on how to achieve healthy and successful aging. A decline in immune function with age is unanimously supported by many epidemiological and clinical observations, with a decrease in T-cell mediated function encompassing a large part of this alteration. In the EU-funded VITAGE project, the effects of aging on biomarkers of immune status are being studied in three European countries. According to strict inclusion/exclusion criteria, a cohort of 300 healthy male non-smoking 20–75 years old volunteers were enrolled in France (n = 99), Spain (n = 100) and Austria (n = 101). In each country, the volunteers were classified as a function of age (one age group per decade). Biomarkers of immune status were determined including delayed-type hypersensitivity tests, measurement of lymphocyte surface markers, and serum determinations of interleukin-2, complement fractions and immunoglobulins.

**Results:**

There were moderate differences in the biomarkers of immune status of the VITAGE study volunteers among the three European centres. The percentage of Natural Killer (NK) cells was 156% and 142% higher in Spain as compared to France and Austria, respectively (p < 0.0001), and this increase was observed at any age group above 30 years. Comparison between age-groups showed that in Spain, but not in France or Austria, older individuals had significantly a lower B lymphocyte distribution and conversely, a higher NK cell distribution. Moreover, the CD4/CD8 ratio was positively correlated with age in Austrian subjects (p < 0.0001).

**Conclusion:**

Our results provide evidence of an increased NK cell distribution in the elderly, especially in the Spanish population. NK cell status may predict morbidity and mortality in the elderly, emphasizing the importance of innate as well as adaptive immunity in ensuring healthy longevity and cancer resistance, possibly in link with the Mediterranean diet.

## Introduction

The mechanisms by which a successful aging occurs in humans, i.e. aging in good psychophysical conditions [1], are immunologically characterized by preserved lymphoproliferative responses and Natural Killer (NK) cell cytotoxicity as well as conserved antigen presentation (reviewed in [2,3]). BELFAST nonagenarians show evidence of a competent immune system, programmed with increase in the number and/or the proportion of NK cells to scan presumably for virus-infected, stressed and malignant cells [4]. In others studies, an increase in NK cells showing a mature phenotype was found in healthy elderly donors who had an NK cytotoxic capacity of total peripheral blood lymphocytes preserved [5,6]. Such preservation in healthy elderly may be due to a high number of NK cells in order to compensate low NK cell cytotoxicity and cytokine and chemokine production [7].

The decline in immune function with age is unanimously recognized and supported by epidemiologic and clinical studies [8]. We have reported that several metabolic and nutritional factors, including insulin, retinoic acid or carotenoids affect the immune response in function of age in healthy volunteers [9-13]. However, the development of age-related changes in health status in elderly men have led to conflicting results and these discrepancies may be partially ascribed to the population tested, i.e. presence of concomitant pathologic disorders or nutritional deficiencies that may affect the immune status (reviewed in [14-17]). To overcome this problem, volunteers in the present study were recruited according to the SENIEUR protocol, which used strict clinical and biological inclusion criteria (i.e. good nutritional status, absence of disease, no sign of inflammation or infection, no drug delivery) as well as rigorous exclusion criteria with detailed measurement of the immune markers [18].

In Europe, the prevalence and the geographical variation of symptoms associated with a putative “aging syndrome” have never been documented. A recent multi-centre study was designed from male volunteers to specify age-related changes in hormone levels, socioeconomic and lifestyle factors that exist across Europe [19]. However, the effects of aging on biomarkers of immune status, measured at baseline in healthy individuals from different European countries, have never been reported. The geographical comparisons are of particular interest, since in addition to the immunological status, various potential predisposing risk factors such as the dietary, lifestyle habits, genetic and socioeconomic factors are different from a country to another [20,21].

The aim of the present work was to provide descriptive information on the biomarkers of immune status in 300 healthy volunteers aged between 20–75 years recruited in Austria, Spain and France and having participated in our previous EU-funded studies [22-25]. The number of about 100 subjects in each country has been chosen on the basis of the results of power calculations for those biomarkers with known distribution in humans, and assuming to detect biologically significant differences in each case. The decision to limit this project only to male subjects has been based on the observation that changes in hormonal status that occur in women, both within and between subjects of this wide age range, may affect the immune status [26].

Our preliminary and primarily descriptive analyses showed that there are moderate differences in the immune status of middle-aged to older inhabitants of the three European centres. Moreover, blood leukocyte phenotypes, especially NK and CD8 cell distribution, were markedly increased in elderly volunteers from the Spanish centre, thus suggesting a specific immune pattern possibly in link with environmental factors such as the Mediterranean diet and the lifestyle.

## Results

### Determination of systemic biomarkers of immune status

**Figure 1:**
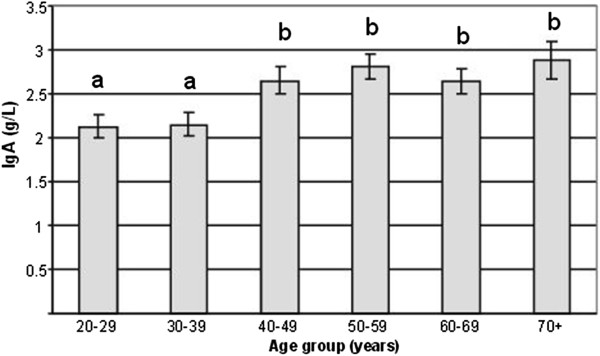
**IgA serum levels in function of age of the volunteers from the 3 countries.** Results are presented as means ± SEM. Two-way ANOVA was performed to discriminate among the two factors age-group, country and their interaction followed by a post-hoc Newman-Keuls test. A significant age group-related effect was observed independently of the country-related factor. Means sharing different superscript letters are significantly different: a≠b≠c, p < 0.05.

The measurements of all immune biomarkers tested (IgG, IgA, IgM, C3, C4, sIL-2R) were in the usual physiological range observed in others European countries [27,28] and there was no biological difference from a country to another (Table 1). In France, Spain and Austria, individuals had IgA levels positively correlated with age (r^2^ = +0.2, p = 0.04). Accordingly, an age effect was found for IgA serum levels which were significantly higher in subjects aged 40 years old or more compared to younger individuals (Figure 1). Comparison between age groups showed no difference among the others serum immune biomarkers tested (IgG, IgM, C3, C4, sIL-2R, data not shown).

**Table 1:** Baseline immune parameters for the 3 populations

	**France**	**Austria**	**Spain**	**Total**
	**(n = 99)**	**(n = 101)**	**(n = 100)**	**(n = 300)**
**IgG (g/l)**	11.1 ± 0.2	11.0 ± 0.2	11.1 ± 0.3	11.1 ± 0.1
**IgA (g/l)**	2.60 ± 0.10	2.20 ± 0.10	2.72 ± 0.12	2.50 ± 0.06
**IgM (g/l)**	1.20 ± 0.06	1.00 ± 0.04	1.18 ± 0.07	1.10 ± 0.03
**C3 (g/l)**	1.00 ± 0.02	1.00 ± 0.01	1.09 ± 0.02	1.00 ± 0.01
**C4 (g/l)**	0.20 ± 0.01	0.20 ± 0.01	0.25 ± 0.01	0.20 ± 0.00
**sIL2-R (pmol/l)**	38.3 ± 1.7	36.0 ± 2.1	39.3 ± 2.0	38.8 ± 1.4

Means ± SEM.

Two-way ANOVA was performed to discriminate among the two factors age-group, country and their interactions. No interaction was obtained between the two factors and no effect was observed whatever the factor.

### Determination of delayed-type hypersensitivity

Considering the delayed-type hypersensitivity (DTH) responses, the cumulative score and the number of positive reactions were higher in Austria (22.7 ± 1.2 and 6.9 ± 0.1, respectively) than in France (17.7 ± 0.9 and 3.4 ± 0.2, respectively) and in Spain (7.1 ± 0.8 and 1.1 ± 0.1, respectively) (Table 2). For both Austria and France, the cumulative score was in the normal range while it was slightly hypoergic in Spain as defined by Knicker et al. [29]. In each of the three countries, the number of positive responses was more frequent for tetanus and tuberculin. In Spanish volunteers, a positive correlation was found between age and the cumulative score (p = 0.04). Accordingly, the cumulative score was significantly lower in the age group 20–29 y (2.6 ± 0.9) compared to the oldest age groups 40–49 y (8.7 ± 1.7), 50–59 y (8.3 ± 2.2) and 60–69 y (9.4 ± 2.3). In French and Austrian volunteers, no correlation was found between age and either the number of positive reactions or the cumulative score (data not shown).

**Table 2:** Delayed-type hypersensitivity in the 3 populations

	**France**	**Austria**	**Spain**	**ANOVA**
	**(n = 99)**	**(n = 101)**	**(n = 100)**	
**Number of positive reactions**	**3.4 ± 0.2**^**a**^	**6.9 ± 0.1**^**b**^	**1.1 ± 0.1**^**c**^	**<0.0001**
**Cumulative score (mm)**	**17.7 ± 0.9**^**a**^	**22.7 ± 1.2**^**b**^	**7.1 ± 0.8**^**c**^	**<0.0001**
Proteus mirabilis	2.4 ± 0.2^a^	3.2 ± 0.2^b^	0.6 ± 0.2^c^	**<0.0001**
Trichophyton	0.9 ± 0.1^a^	1.4 ± 0.2^b^	0.2 ± 0.1^c^	**<0.0001**
Candida albicans	2.3 ± 0.2^a^	2.7 ± 0.3^a^	0.4 ± 0.1^b^	**<0.0001**
Tetanus	4.2 ± 0.3^a^	6.4 ± 0.4^b^	2.5 ± 0.3^c^	**<0.0001**
Diphteria	1.5 ± 0.2^a^	2.8 ± 0.4^b^	0.6 ± 0.2^c^	**<0.0001**
Streptococcus	0.9 ± 0.2^a^	1.3 ± 0.2^b^	0.2 ± 0.1^c^	**<0.0001**
Tuberculin	5.4 ± 0.3^a^	4.5 ± 0.5^b^	2.7 ± 0.4^c^	**<0.0001**

Mean ± SEM.

Two-way ANOVA was performed to discriminate among the two factors age-group, country and their interactions followed by a post-hoc Newman-Keuls test. A significant country-related effect was observed independently of the age-group: a≠b≠c, p < 0.0001.

### Determination of lymphocyte phenotypes

#### ***CD4 and CD8 T cell distribution***

**Figure 2:**
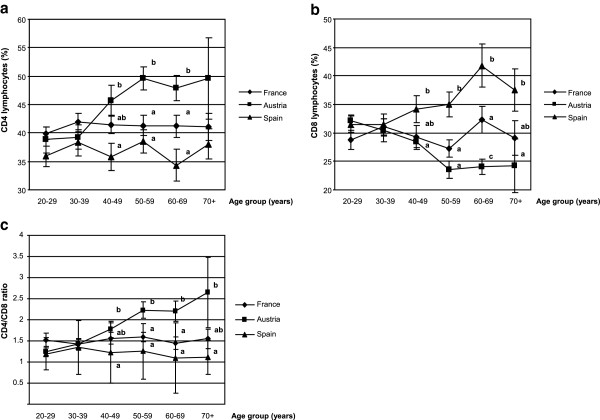
**T lymphocyte distributions and ratio.** CD4 **(a)** and CD8 **(b)** lymphocyte distributions and CD4/CD8 ratio **(c)**. T lymphocyte distribution and CD4/CD8 ratio were compared for each age group between the three countries (A = Austria; F = France; S = Spain). Data are reported as means ± SEM, one way ANOVA followed by a Newman-Keuls test, different superscript letters indicate a statistical difference between country (a≠b≠c, p < 0.05).

The proportion (in %) of total lymphocytes was not different either between the three countries (Table 3) or between the age groups for each country (data not shown). However, the distribution of CD4 lymphocytes was significantly higher in Austria (44.0 ± 1.1) than in France (41.1 ± 0.7) or in Spain (36.7 ± 0.9) while the opposite was observed for CD8 lymphocytes whose percentage was higher in Spain (34.8 ± 1.1) than in France (29.7 ± 0.8) or in Austria (27.7 ± 0.7) (Table 3). These findings were markedly observed in volunteers aged 40 years old or more (Figure 2). The CD4/CD8 ratio was significantly different in Austria (1.8 ± 0.1), in France (1.5 ± 0.1) and in Spain (1.2 ± 0.1). In Austrian volunteers, age was positively correlated with CD4 cells distribution (r^2^ = +0.356, p = 0.0002), with the CD4/CD8 ratio (r^2^ = +0.452, p < 0.0001) and was negatively correlated with CD8 cell distribution (r^2^ = -0.449, p < 0.0001). Moreover, age was positively correlated with CD8 cell distribution in Spanish subjects (r^2^ = +0.338, p = 0.0005).

**Table 3:** Lymphocyte phenotype distribution (%) for the 3 populations

	**France**	**Austria**	**Spain**	**ANOVA**
	**(n = 99)**	**(n = 101)**	**(n = 100)**	
**T Lymphocytes**				
**Total**	**70.2** ± **0.6**	**70.5** ± **1.1**	**67.2** ± **1.1**	**NS**
CD4	41.1 ± 0.7^**a**^	44.4 ± 1.1^**b**^	36.7 ± 0.9^**c**^	**<0.0001**
CD8	29.7 ± 0.8^**a**^	27.7 ± 0.7^**a**^	34.8 ± 1.1^**b**^	**<0.0001**
CD4/CD8	1.5 ± 0.1^**a**^	1.8 ± 0.1^**b**^	1.2 ± 0.1^**c**^	**<0.0001**
**B Lymphocytes**	**13.7** ± **0.4**^**a**^	**16.0** ± **0.7**^**b**^	**13.3** ± **0.8**^**a**^	**0.01**
**NK cells**	**14.8** ± **0.7**^**a**^	**16.2** ± **0.7**^**a**^	**23.1** ± **1.0**^**b**^	**<0.0001**

Means ± SEM.

Two-way ANOVA was performed to discriminate among the two factors age-group, country and their interaction followed by a post-hoc Newman-Keuls test. A significant country-related effect was observed independently of the age-group: a≠b≠c, p < 0.01, NS: not significant.

#### ***B and NK cell distribution***

**Figure 3:**
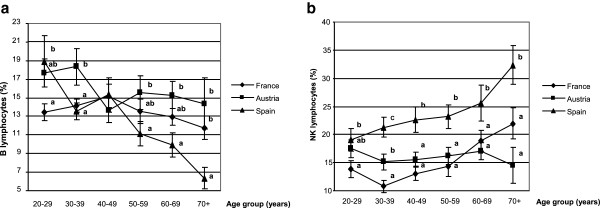
**B and NK lymphocyte distribution.** B **(a)** and NK **(b)** cell distribution were compared for each age group between the three countries (A = Austria; F = France; S = Spain). Data are reported as means ± SEM, one way ANOVA followed by a Newman-Keuls test, data that share different superscript letters indicate a statistical difference between country (a≠b≠c, p < 0.05).

The lowest percentage of B lymphocytes was observed in the Spanish individuals (Table 3), especially in those above 50 years old (Figure 3a). Conversely, the percentage of NK cells was significantly higher in Spain (23.1 ± 1.0) than in France (14.8 ± 0.7) or Austria (16.2 ± 0.7) (Table 3). This increase was observed in the Spanish population at any age group over 30 y (Figure 3b). Age was negatively correlated with B cell distribution in Spanish volunteers (r^2^ = -0.436, p < 0.0001). In contrast, age was positively correlated with NK cell distribution in Spanish (r^2^ = +0.345, p = 0.0004) and French (r^2^ = +0.349, p = 0.0004), but not in Austrian subjects.

**Figure 4:**
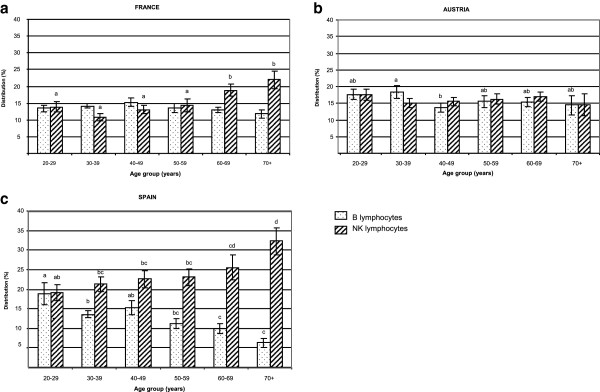
**B lymphocyte and NK cell distribution.** The B and NK cell distribution was compared for each age group in each country. Statistical analysis was done by one way ANOVA followed by a Newman-Keuls test; p < 0.05 was considered significant and data with different superscript letters are significantly different (a≠b≠c≠d).

Comparison between age groups showed that B cell distribution was significantly impaired in elderly Spanish subjects over 60 years old (Figure 4c). The same age cut-off (60 y) was observed for NK cell increase in Spanish and French (Figure 4c and a) but not in Austrian subjects (Figure 4b).

#### ***CD45RA and CD45RO CD4 lymphocyte distribution***

**Figure 5:**
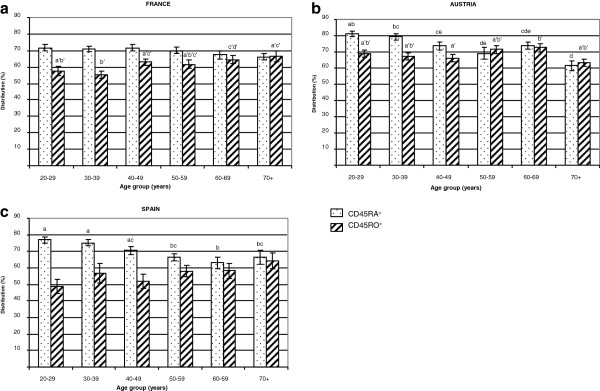
**CD45 lymphocyte distribution.** Naïve (CD45RA^+^) and memory (CD45RO^+^) CD4^+^ lymphocyte distribution was compared for each age group in each country. Values are means ± SEM. Statistical analysis was done by one way ANOVA followed by a Newman-Keuls test, p < 0.05 was considered significant. Values with different superscript letters are significantly different (a≠b≠c≠d≠e; a'#b'#c').

CD45RA and CD45RO CD4 cell distributions (in %) were markedly higher in Austria (74.9 ± 1.1 and 68.8 ± 1.0, respectively) than in France (70.1 ± 0.8 and 60.8 ± 0.1, respectively) or Spain (70.3 ± 1.1 and 55.4 ± 1.9, respectively). Distribution between naïve (CD45RA) and memory (CD45RO) CD4 cells was modified by aging (Figure 5). As expected, percentages of CD45RA CD4 were higher in young adults than in elderly subjects, and conversely, percentages of CD45RO CD4 cells were lower in young adults than in older subjects. Age was negatively correlated with CD45RA CD4 cell distribution in Austria (r^2^ = -0.376, p = 0.0001) and Spain (r^2^ = -0.430, p < 0.0001) but not in France. On the other hand, age was positively correlated with CD45RO CD4 cell distribution only in France (r^2^ = +0.289, p = 0.0036).

#### ***CD11b and CD18 PMN distribution***

Adhesion antigens (CD11b, CD18) expression (in %) of polymorphonuclear neutrophils (PMNs) was not affected, neither by the geographical location (France: 99.5 ± 0.1; Austria: 99.9 ± 0.02; Spain: 99.9 ± 0.01 for CD11b and France: 99.8 ± 0.1; Austria: 99.9 ± 0.02; Spain: 99.9 ± 0.01 for CD18), nor by the age of the volunteers (data not shown).

## Discussion

The purpose of this work was to determine the biomarkers of immune status in healthy volunteers and to provide a geographical comparison of these biomarkers between three European countries. Despite different dietary habits, lifestyle, genetic and socio economic factors, we found only moderate variations in the biomarkers, the most striking being an increased distribution of NK cells in Spain, compared to France and Austria, while a higher DTH response and an enhanced CD4/CD8 ratio were found in Austria. Interestingly, the immune parameters in our French volunteers had constantly intermediate values between those measured in Austrian and Spanish individuals, thus reflecting the importance of a geographic variability on the immune status and possible gradual differences in the dietary, lifestyle habits, environmental, genetic and socio-economic factors between Southern and Northern Europe. Immunosenescence affects all compartments of the immune system. Age changes were demonstrated in T lymphocytes and in innate immunity, including NK cells. A decline in CD4 count, a rise in CD8 compartment and an increase in the NK cell numbers with well-preserved [30] or reduced cytotoxic function [31,32] has been considered as a “remodelling” of the immune compartment with aging [30].

### NK cell status and longevity

In our Spanish population (mean age 46.3 y), we observed an increase in the percentage of NK cells in elderly people when compared to young controls (32.3 ± 3.4 in elderly over 70 y *vs* 19.1 ± 2 in young aged 20 to 29 y). This is consistent with the findings of Borrego *et al.* who also observed a significant increase in the percentage of CD56^+^ cells in aged Spanish donors when compared to the young controls (29.1 ± 10.1 in elderly over 81 y *vs* 14.8 ± 5.3 in young aged 32 y) [33]. Moreover, we found that the percentage of NK cells was significantly higher in Spanish than in French or Austrian subjects, and this was observed at any age group above 30 years. The NK cell status is thought to be an important component of the aging immune system [5,7] and can predict morbidity and mortality in the elderly. Low NK cell number and function are associated with increased mortality and greater risk of severe infections in elderly subjects [34,35]. Conversely, a well preserved functional status of NK cells until very advanced age is linked to healthy ageing and longevity [5], emphasizing the importance of innate as well as adaptive immunity in ensuring healthy longevity [30] and possibly cancer resistance [36,37]. Thus, our results may be related to the fact that life expectancy at birth is greater in Spain (over 80 y) as compared to France or Austria (below 80 y) [38].

### Environmental factors and immune biomarkers

An aging-related deficiency in the immunosurveillance system (especially NK cells) may play a role in pathogenesis and in particular, in cancer. Since many environmental factors are mutagenic and promote cancer, we can speculate that aging is clearly related to the duration of exposure to these factors and their effects on immune system. For example, pollution affects the number of NK cells but also of CD4^+^, CD8^+^ and B lymphocytes [39]. As such, we cannot rule out that the differences in lymphocyte distribution observed between the three countries may be explained by differential environmental factors such as pollution or others.

### Impact of diet on immune aging

As the consumption of fruits and vegetables is higher in Spain than in the other European countries [40,41], an important role for the dietary patterns in the healthy aging of the elderly has been suggested [42,43]. In Europe, prospective studies have shown that adherence to a typical Mediterranean diet is associated with lower mortality and increased longevity [41,44-46]. The effects of such a diet on immune status have not been reported. In one recent study conducted in patients with high risk of cardiovascular diseases, the Mediterranean diet, compared to a control low-fat diet, seems to down-regulate immune cell activation but not T lymphocyte percent change [47]. In the meta-analysis of 12 prospective studies [48], a two-point increase in a score of Mediterranean diet adherence was associated with improved health status, as shown by a significant reduction in mortality (9%), cardiovascular diseases (9%), cancer (6%), and the incidence of Parkinson’s and Alzheimer’s disease (13%). However, as this benefit was also apparent in non-Mediterranean populations [47,48], a direct role of diet on aging remains unclear and cannot fully explain our results. NK cells and their cytolytic activity have also been related to nutrition including vitamin D and anthropometric markers [49]. Preliminary results from an Irish study indicate a small negative relationship between NK cell number and body mass index [4].

### Age effect on immune status

In our well-defined healthy population, we found serum IgA levels were correlated with age, which is in agreement with other studies [27] and suggests either a complex derangement of B cell function with age [50] or a remodelling of the immune system rather than a deterioration [51]. Comparison between age groups showed that IgA levels were higher in subjects above 40 y. It is noteworthy that the same cut-off was observed for other immune biomarkers (B, CD4 and CD8 cell distribution, CD4/CD8 ratio), suggesting that a decline in immune function leading to the healthy aging process may start in the early age. By recruiting only male volunteers in our study, we have excluded any possible influence of immune status by hormonal changes that occur with age in women. We also found alterations in the immune capacity reflected by a greater proportion of NK cells, a decrease in B lymphocytes but a stable number of T cells. When all European volunteers were classified as a function of age, NK distribution was significantly increased with age (17% in 20–29 y *vs* 24% in 70 y), in agreement with previous findings ([52]: 18% in 29 y *vs* 30% in 86 y; [53]: 14% in 20–29 y *vs* 20% in 70–75 y). Although the alterations of NK cells and other innate immune cells with aging are generally less marked than those seen with T cells [16,54], we observed that in our subjects, a greater correlation with age was found for NK cells (r^2^ = +0.21, p = 0.0002) than for CD4/CD8 ratio (r^2^ = +0.14, p = 0.01). A similar positive correlation between age and CD4/CD8 ratio in Irish individuals was reported by Rea et al. [4]. Moreover, we observed a shift from a predominance of naïve T cells towards T cells expressing memory phenotypes from the youngest to the oldest age groups, as previously reported [12,55]. This shift may result from the age-related repetition of antigenic challenges.

### Vaccination and immune response

The mechanisms of vaccination are complex and may differ depending on the allergen and the route of immunization. We observed that the most frequent DTH responses were observed for tetanus and tuberculin which represent two common antigens. As the vaccination coverage against the different antigens used in our study has not been reported in Europe, it is unclear why we observed enhanced DTH responses in our Austrian volunteers, as compared to the subjects from the other two European centres. Whether this finding could be explained by a better vaccination status or linked to booster doses in elderly people in Austria is unlikely. Based on the influenza vaccination coverage rate in the general population (aged 14 to 75 years), it was of 26.4% in France and 23.7% in Spain, during the period 2007-2008 [56]. By comparison, the vaccination coverage rate during the same period was significantly lower in Austria (16.0%), where vaccination is costly and results in socioeconomic inequalities regarding immunization in the general population [57]. In addition, adherence to vaccination programs may differ between European countries. For example, tuberculin skin testing to make decision about BCG revaccination was described to be routinely performed in France but not in Austria [58]. Moreover, independently of the vaccination policy, the low cutaneous reactivity in Spain might be in part linked to dietary habits particularly fruits and vegetables consumption as previously published [59].

## Conclusion

This study compares for the first time the immune status in three European countries and describes the effects of aging on immune cell distribution. The differences observed from a country to another can be explained by genetic, environmental, dietary and socioeconomic variations. This variability should be taken into account when estimating, in future nutrition research studies, the actual immune status of healthy individuals with different geographical origins.

## Methods

### Subjects and experimental procedure

Three-hundred healthy male non-smoking subjects, aged 20–75 years (stratified by age), were recruited in Clermont-Ferrand, France (n = 99), Graz, Austria (n = 101), and Reus, Spain (n = 100), as part of the European Commission-funded Research and Technology Development (RTD) project of the 5th Framework Program, specific RTD Program “Quality of Life and Management of Living Resources”, Key Action#1, “Food, Nutrition, and Health”, entitled “Vitamin A, Vitamin E, and Carotenoid Status and Metabolism during Ageing: Functional and Nutritional Consequences”, acronym VITAGE (Contract QLK1-CT-1999-00830) [22,23]. The study protocol was approved by the local Ethics Committee of the three recruiting centres and was performed in accordance with the ethical standards of the Declaration of Helsinki. A written informed consent was obtained from all participants prior to their inclusion in the study. After informative sessions, a trained medical doctor conducted a personal interview to obtain information on anthropometric measurements, personal history, lifestyle, use of medications, physical activity, smoking habits, and use of dietary supplements containing vitamins or trace elements. Exclusion criteria were familial hypercholesterolemia, chronic diseases (including diabetes, cancer, cardiac insufficiency, neurological diseases, inflammatory diseases and chronic diseases of the liver, lung, or thyroid, non stable hypertension, dementia, and infectious diseases known to affect the immune system, such as human immunodeficiency virus and hepatitis C), vaccination during the past 2 months, alcoholism or drug addiction, competitive sport activities, and the consumption of special diets or dietary supplements in the past 3 months. All volunteers from a given centre had the usual diet of their respective country, which is the diet of the general population. Venous blood samples were collected from subjects in the fasting state for serum and leukocyte isolation and storage until analysis. The SENIEUR protocol standardized the selection of the volunteers for immunological studies [18].

The mean age ± SEM of the volunteers from France, Austria and Spain was 46.8 ± 1.5 y, 45.6 ± 1.5 y and 46.3 ± 1.6 y, respectively (no significant difference). In each country, the volunteers were classified in six different age groups: 20–29 y (n = 59), 30–39 y (n = 55), 40–49 y (n = 59), 50–59 y (n = 51), 60–69 y (n = 56) and 70–75 y (n = 20). This approach allowed us either to directly compare two age groups or to describe the evolution of the study variables as a function of age.

### Determination of systemic biomarkers of immune status

Serum immunoglobulin (IgG, IgA, IgM) and complement fraction (C3, C4) concentrations were quantified by immunonephelemetry (Array protein system, Beckman-Coulter, Villepinte, France), using human antibodies (Beckman-Coulter). Values are expressed in g/l.

Serum soluble interleukin 2 receptor (sIL-2R) concentration (pmole/l) was assayed by ELISA (Immunotech kit, Beckman-Coulter).

### Determination of delayed-type hypersensitivity skin test response

To determine DTH skin response, we used the Multitest CMI® skin applicator (Pasteur-BioMérieux, Lyon, France) against seven antigens (Tetanus, Diphteria, Streptococcus (C group), Tuberculin, *Candida Albicans*, Trichophyton, *Proteus Mirabilis*) and a glycerin negative control. Multitest® was applied on healthy arm skin for at least 5 seconds. The skin tests were read at 48 h after application by measuring for each antigen the induration response (mean of two perpendicular diameters) using a gradual scale. Induration ≥2 mm was recorded as positive response and corrected by substracting the negative control, if any [60]. The cumulative score was calculated as the sum of all antigen indurations. This score was considered as “hypoergic” (<10 mm with at least one positive response ≥2 mm) or “anergic” (no response greater than 2 mm) or “normal” (≥10 mm with at least one positive response ≥2mm) according to Kniker et al. [29].

### Determination of blood leukocyte phenotype

In each recruitment centre, blood leukocytes were isolated using Ficoll density gradient (Histopaque®1077 and 1119, Sigma-Aldrich) and then cryopreserved [61]. Briefly, cells were progressively frozen in a Cryo-Med liquid nitrogen freezer and conserved at -196°C during at most 6 months. Such cryopreservation did not affect the cell surface markers [62]. All analyses were done in the same laboratory: lymphocyte subpopulations were measured, as previously described [13], using flow cytometry with an Epics XL (Beckman-Coulter) after labelling with fluorochrome-conjugated monoclonal antibodies (Beckman-Coulter) : CD3-PC_5_, CD4-RD_1_, CD8-ECD, CD45RA-FITC, CD45RO-FITC, CD19-ECD, CD56-PE (Beckman-Coulter) corresponding to total T cells (CD3^+^), T helper/inducer (CD4^+^), T suppressor/cytotoxic (CD8^+^), B lymphocytes (CD19^+^) and NK cells (CD56^+^), respectively. Results were expressed in percentages of total leukocyte populations. PMN CD expression was determined by flow cytometry using anti-CD11b-FITC and anti-CD18-FITC (Beckman-Coulter) and reported in percentage of total PMN population. Appropriate controls i.e. conjugated isotypes and compensation settings in case of multiple labellings were done and intra- and inter-assays were recorded as previously reported [13].

### Statistics

All statistical analyses were run on Statview SAS, version 5. The experimental design comprised two cross-fixed factors with the factor “aging” as six groups and the factor “country” as three groups. This design allowed statistical analysis by two-way measures analysis of variance (ANOVA) in order to discriminate between the age effects and the country-related effects. The level of significance was set at p ≤ 0.05 for this test. When the ANOVA indicated significant interactions, the Newman-Keüls post-hoc test was used to identify differences between individual means. When no significant interaction was found, the marginal means were calculated and compared with a one way ANOVA followed by a Newman-Keüls test. Results are expressed as means ± SEM and various superscript letters (a, b, c, d, e) are significantly different (p < 0.05). To assess the correlations between baseline immune parameters and age, Spearman’s rank correlation tests was used for dependent groups and the Kruskal–Wallis test was performed for independent groups.

## Competing interests

The authors declare that they have no competing interests.

## Authors’ contributions

The authors’ responsibilities were as follows: M-PV, JR, BW-R and ER: coordination and design of the experiments; M-PV, M-CF, NG-M, JT, ER and AR: collection and analysis of data; M-PV: writing of the manuscript and provision of significant advice. All authors read and approved the final manuscript.

## Supports

This study was carried out with financial support from the Commission of the European Communities, specific RTD program “Quality of Life and Management of Living Resources”, QLK1-CT-1999-00830, VITAGE.
